# Exploring the Efficacy of Computer-Assisted Navigation in Improving Lag Screw Placement and Preventing Cut-Out in Intramedullary Nail Fixation of Femoral Fractures: A Meta-Analysis

**DOI:** 10.7759/cureus.77724

**Published:** 2025-01-20

**Authors:** Peter Richa, Jason S DeFrancisis, Victoria L Young, Feross Habib, Paul Danahy

**Affiliations:** 1 Orthopedic Surgery, Lake Erie College of Osteopathic Medicine, Bradenton, USA

**Keywords:** cephalomedullary nail, computer-assisted surgery, cut-out, intramedullary nail, lag screw, lower limb orthopedic surgery, proximal femur fractures, tip-apex distance

## Abstract

Femoral neck fractures are a common complication treated by orthopedic surgeons. Exploring the role of computer-assisted orthopedic programs in femoral fracture treatment is of particular interest given the technological advances in computer-assisted programs in the medical field. Notably, systems such as Stryker’s Adaptive Positioning Technology (ADAPT) may allow for more precision in determining the tip-apex distance (TAD) when treating intertrochanter femur fractures. Such innovations hold the potential to reduce complication rates, including the incidence of lag screw cut-out, which could improve clinical outcomes in intertrochanter femur fracture treatment. This meta-analysis aims to evaluate the effectiveness of computer-assisted orthopedic systems in improving lag screw placement, as determined by the TAD and, ultimately, screw cut-out. Three studies were compared that reported continuous data for TAD in groups that did and did not use Stryker’s ADAPT computer-assisted system. A random effects model was utilized to identify heterogeneity between studies. This was determined by variation and calculated through Cochran’s Q-test, I^2^ statistic, and Tau^2^. Operative time was also reported in these studies and was evaluated as a secondary outcome. Each study analyzed showed that ADAPT had a statistically significant improvement in TAD with an overall effect size of -5.06. However, with an I^2^ value of 89% (p<0.01), there was notable heterogeneity between the three studies compared in this meta-analysis. While it is clear that there are benefits to using computer-assisted technology for internal femur fixation, more research is needed to understand the implications, including operative time and possible improvements in screw position.

## Introduction and background

Proximal femoral fractures are among the most common fractures seen by orthopedic surgeons [1]. In the United States, proximal femoral fractures are estimated to cost the healthcare system over $13.8 billion annually, exceeding $50,000 per case [2]. These fractures can be divided into intracapsular, which involves the femoral neck, and extracapsular, involving the trochanteric and subtrochanteric regions [2]. On the other hand, distal femoral fractures are rare, accounting for less than 0.5% of all fractures [3]. Excluding the immediate complications associated with these fractures, proximal femoral fractures significantly impact an individual’s quality of life and mortality risk [4]. Age, osteoporosis, trauma, falls, gender, and bone density are a few of the numerous risk factors associated with femoral fractures [4]. With the increase in life expectancy and the number of elderly individuals within the population, the need to effectively manage proximal femoral fractures is progressing [1].

The current standard of care for intertrochanteric fractures, which characterize over half of femoral fractures, is internal fixation. Internal fixation is performed utilizing either a sliding hip screw mechanism or a cephalomedullary nail to stabilize the fractured segment [5]. While controversy and discussion may exist about the superiority of either of these two differing techniques, the need to minimize tip-apex distance (TAD) is universal. TAD is identified as the sum of the distance from the lag screw to the apex of the femoral head [5]. Utilizing lateral and anterior-posterior radiographs, orthopedic surgeons strive for central and precise screw placement [6]. If surgical techniques are flawed, a dreaded complication identified as lag screw cut-out may occur [6]. The lag screw cut-out is defined as a mechanical failure in internal fixation due to the intra-articular extrusion through the femoral head as a consequence of a varus collapse of the neck shaft angle [6]. As described by Baumgaertner, TAD is one of the most critical risk factors for lag screw cut-out, and a distance greater than 25 mm significantly increases the risk of such an event taking place [3]. To further support this claim, a study conducted by Geller et al. found that 44% of patients with a TAD exceeding 25 mm experienced lag screw cut-out, whereas none of the patients with a TAD below 25 mm had this complication [7]. The position of the lag crew, fracture reduction, and pathological conditions, such as necrosis of the femoral head, as noted by Gazzoti et al., are a few of the various factors that also affect the incidence of lag screw cut-out [8]. Rehospitalization, bone loss, and conversion to arthroplasty are a few of the many complications that result from screw cut-outs [1].

As technology advances, computer-assisted orthopedic systems have become increasingly popular in attempts to refine surgical techniques and advance outcomes [9]. Computer-assisted surgery generally alludes to surgical techniques that integrate the efforts of both surgeons and machines, which has become widely accepted for use in various orthopedic procedures [10,11]. Systems such as the Adaptive Positioning Technology (ADAPT) computer-assisted system developed by Styrker may be advantageous within the field of orthopedics. The Stryker ADAPT system delivers intraoperative information concerning the implant placement of the Gamma3 nail [12]. These recently introduced systems utilize fluoroscopy to transform two-dimensional X-ray images into three-dimensional concepts [13]. The three-dimensional information aims to allow surgeons to precisely determine the distances from the screw tip to the femoral head surface, previously described as the TAD [13]. The significance of reducing the TAD to prevent complications during osteosynthesis characterizes the importance of utilizing advanced systems within proximal femoral fractures [14]. To date, comprehensive systematic reviews elucidating the efficacy of computer-assisted orthopedics systems in minimizing TAD are very minimal. Given this, our meta-analysis aims to evaluate the effectiveness of computer-assisted orthopedic systems in improving lag screw placement, as determined by the TAD and, ultimately, screw cut-out. In addition, we analyzed the influence of computer-assisted orthopedics systems on the operative time.

## Review

Materials and methods

Research Question

Does the use of computer-assisted navigation minimize TAD and decrease the incidence of lag screw cut-out in intramedullary nail fixation?

*Inclusion Criteria*

The study population includes adult males and females aged 18 years or older who have been diagnosed with an intertrochanteric femoral fracture. The study design encompasses randomized controlled clinical trials, prospective cohort studies, and retrospective cohort studies. The intervention being evaluated is the use of Intramedullary Gamma3 nails with Stryker’s ADAPT system, compared to the use of these nails without computer-assisted navigation. The studies must include a control group for comparison. The primary outcome is the tip-apex distance (TAD), measured via postoperative radiographs, while the secondary outcome is the operative time. Types of studies considered for inclusion are randomized controlled trials, cohort studies, case-control studies, and retrospective studies that assess the use of computer-assisted navigation for femoral fracture fixation. Only studies published in English are considered.

Exclusion Criteria

Non-relevant studies include manuscripts that are unrelated to computer-assisted navigation for intramedullary nail fixation. Excluded study designs are non-randomized controlled clinical trials, case reports, cross-sectional studies, non-randomized trials, qualitative studies, and systematic reviews. Studies published in languages other than English are also excluded. Additionally, studies published before the year 2005 are not considered. Exclusion criteria for outcomes include failure to report tip-apex distance (TAD), lack of follow-up data, and failure to report operative time.

*Search Strategy*

**Figure 1:**
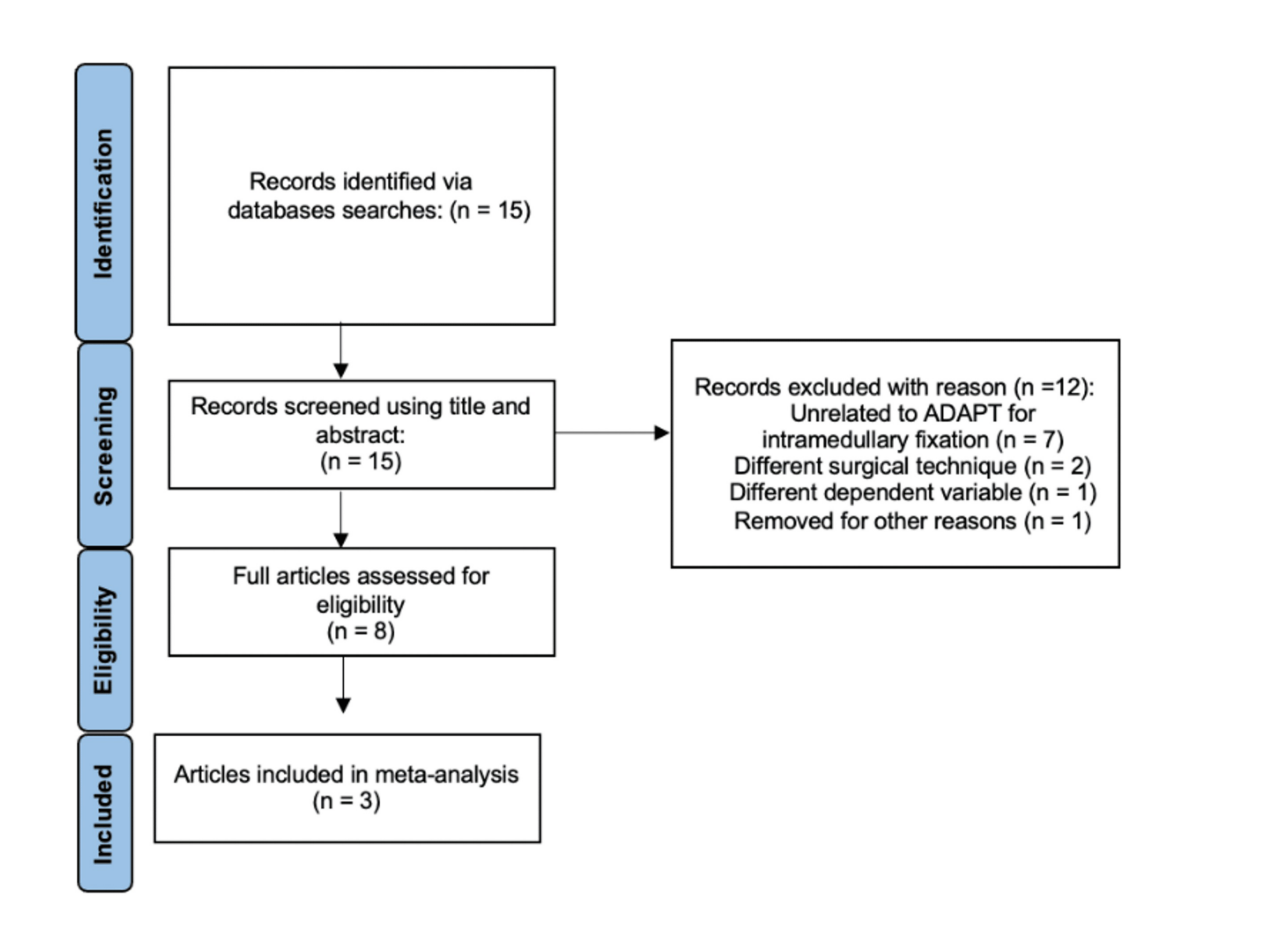
A PRISMA flow chart depicting the literature search and selection process ADAPT: adaptive positioning technology; PRISMA, Preferred Reporting Items for Systematic Reviews and Meta-Analyses

The Preferred Reporting Items for Systematic Reviews and Meta-Analyses (PRISMA) criteria (Figure 1) were utilized for article selection [15]. An extensive database search was performed on titles up to August 2024 in the following databases: PubMed, ScienceDirect, and Cureus. Search terms included “computer-assisted navigation,” “tip-apex distance,” “TAD,” “intramedullary nail fixation,” “ADAPT,” “adaptive positioning technology,” “Gamma3,” “screw cut-out,” “intramedullary nailing,” “femoral fractures,” and “operative time.”

Data Sources

Manual searches of associated meta-analyses and systematic reviews were administered to identify other studies not listed under our database searches.

Study Selection

Non-randomized controlled studies and duplicates were recognized and were not included in the statistical analysis. There were no disagreements among reviewers.

Data Extraction

Data related to study design, sample size, standard deviation, confidence intervals, and tip-to-apex measurements in mm were utilized from each study.

Statistical Analysis

All analyses and graphs were produced utilizing RStudio (v4.4.1, R Core Team, 2024) and the meta package version 7.0-0. A forest plot was generated to present each study’s mean differences and confidence intervals, providing an overall effect estimate. Furthermore, the random effects model, through the incorporation of 95% confidence intervals, was employed to identify heterogeneity between studies. Tests for heterogeneity were performed using Cochran’s Q-test, I2 statistic, and Tau^2^ to analyze variation among studies.

Publication Bias

Publication bias may have limited our search as only studies related to the use of computer-assisted navigation in identifying the TAD of intramedullary nail fixation were included.

Results

Literature Search

The total number of studies found was 15. After filtering for duplicates, unrelated studies, and studies failing to utilize computer-assisted navigation, three articles were included [13,16,17].

Characteristics of Studies Included

Three studies [13,16,17] were included in this meta-analysis, and the characteristics of each are summarized in Table 1.

**Table 1:** Characteristics of studies included in statistical analysis RCS: retrospective cohort study; PCCS: prospective comparative cohort study

Study	Total Sample Size (N)	Design	Treatment	Effects Measured	TAD Units Measured
Murakami et al., 2021 [13]	40	PCCS	Intramedullary nail fixation of femoral trochanteric fractures	TAD, operative time, fluoroscopy time	mm
Herzog et al., 2019 [16]	71	RCS	Intramedullary nail fixation of femoral trochanteric fractures	TAD, operative time	mm
Simcox et al., 2021 [17]	82	RCS	Intramedullary nail fixation of femoral trochanteric fractures	TAD, operative time, fluoroscopy time, lag screw position, radiation dose	mm

*Findings*

**Figure 2:**
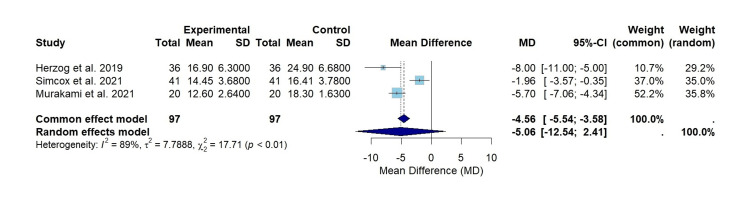
A forest plot of TAD assessment comparing ADAPT treatment versus control treatment from Herzog et al., Simcox et al., and Murakami et al. Experimental: ADAPT treatment group; studies included: Murakami et al., Herzog et al., and Simcox et al. [13,16,17]. TAD, tip-apex distance; ADAPT, Adaptive Positioning Technology

The results for common and random effect models in this manuscript can be found in Figure 2.

A total of three studies provided continuous data for TAD, identified as the sum of the distance from the tip of the screw to the apex of the femoral head on anteroposterior and lateral views [5]. All three studies showed a statistically significant improvement in TAD across the patient cohorts [13,16,17]. The overall effect size for all the studies was found to be mean difference (MD)=-5.06 (95% confidence interval (-12.54, 2.41)), indicating that ADAPT computer-assisted navigation minimized the TAD (Figure 2). The overall I2 value of 89% and p-value <0.01 suggest substantial heterogeneity; therefore, the hypothesis of no heterogeneity may be rejected (Figure 2). These results stipulate that the studies utilized in this analysis had substantially varying effect sizes [18]. The differences may be due to population characteristics or overall methods employed within each study [18].

Figure 2 displays that Murakami et al. had the largest effect size, indicating the greatest magnitude in reducing TAD utilizing the ADAPT system [13]. The effect sizes varied among the studies, as evidenced by the substantial heterogeneity value (I2=89%) (Figure 2). Murakami et al. presented the most significant weight in both the random and common effect models, at 35.8% and 52.2%, respectively (Figure 2) [13]. The size of the box, which represents the weight, signifies this finding. Herzog et al., on the other hand, displayed the lowest precision as the confidence interval exhibited the most extensive range [16].

**Figure 3:**
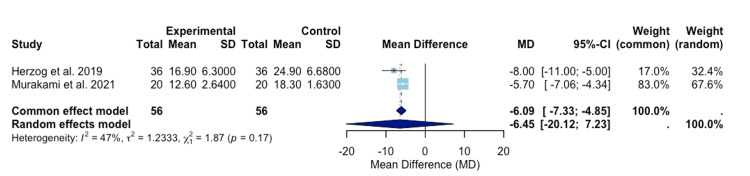
A forest plot of TAD assessment compares ADAPT treatment versus control treatment for Herzog et al. and Murakami et al. Experimental: ADAPT treatment group; studies included: Murakami et al. and Herzog et al. [13,16]. TAD, tip-apex distance; ADAPT, Adaptive Positioning Technology

In Figure 3, a meta-analysis run by Herzog et al. and Murakami et al. computed the heterogeneity for the two studies to be 47% [13,16]. This lower heterogeneity value indicates that less variation exists among these two studies [18]. The mean difference for the common and random effect models of these two studies are -6.09 (-7.33; -4.85) and -6.45 (-20.12; 7.23), respectively (Figure 3), [13,16]. This analysis indicates that the ADAPT system minimized the mean TAD.

**Figure 4:**
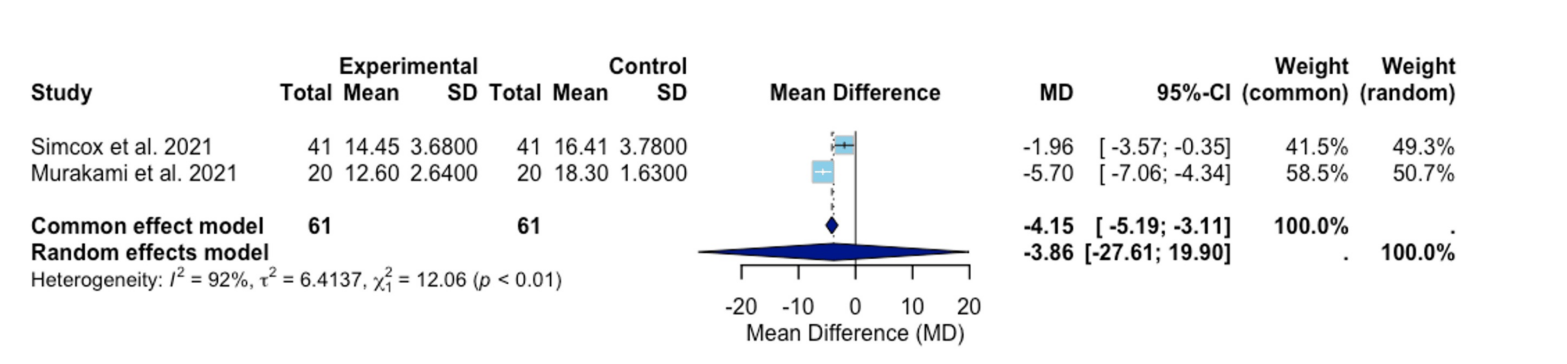
A forest plot of TAD assessment compares ADAPT treatment versus control treatment for Simcox et al. and Murakami et al. Experimental: ADAPT treatment group; studies included: Murakami et al. and Simcox et al. [13,17]. TAD, tip-apex distance; ADAPT, Adaptive Positioning Technology

In Figure 4, a meta-analysis run by Simcox et al. and Murakami et al. identified the heterogeneity between these two studies as 91% [13,17]. This value, higher than the I2 for the analysis of all three studies, indicates a significant degree of variation exists between these two studies [18]. The mean differences for the common and random effect models are -4.15 (-5.19; -3.11) and -3.86 (-27.61; 19.90), respectively (Figure 4) [13,17].

**Figure 5:**
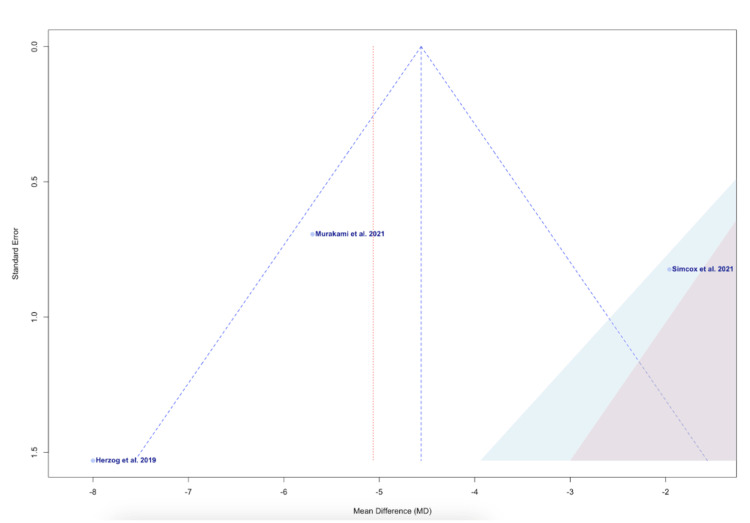
A funnel of publication bias for Herzog et al., Simcox et al., and Murakami et al. Studies included: Murakami et al., Herzog et al., and Simcox et al. [13,16,17].

The funnel plot visualized in Figure 5 assesses the risk of bias across the individual studies [13,16,17]. Murakami et al., the closest study to the midline, represents the lowest risk of bias or variability (Figure 5) [13]. Herzog et al., marking the lowest data point on the plot, represent the study with the smallest effect size (Figure 5) [16].

Discussion

This study explored the effectiveness of computer-assisted navigation in minimizing TAD in patients undergoing intramedullary nail fixation for femoral fractures. Femoral fractures are a very common type of osteoporotic fracture among the elderly and are associated with significant morbidity and mortality [19]. To reduce possible associated comorbidities and complications, such as infection, operative blood loss, and reoperation, it is essential to appropriately secure the proximal fracture [19]. Additionally, surgical intervention of hip fractures should not be delayed; patients with medical comorbidities and delayed surgery have an increased mortality rate [20].

Proximal femoral plating, intramedullary nail fixation, and hip arthroplasty are the various types of surgical procedures utilized to manage this devastating skeletal injury [21]. The study performed by Madboh et al. indicated that intramedullary nail fixation is the preferred method for managing proximal femoral fractures, as the mortality rate was lower and the post-operative functional hip scores were greater for patients who underwent this surgical technique [21].

The most commonly reported complication of intramedullary nail fixation of proximal femoral fractures is lag screw cut-out, as previously described [3]. Due to the positive correlation associated with TAD and lag screw cut-out, minimizing TAD may be crucial in limiting this complication [22]. The results from our meta-analysis, including the total populations for all three studies, displayed a reduction in TAD for intramedullary nail fixation that utilized Stryker’s ADAPT computer-assisted navigation, with an average TAD distance 5.06 mm lower than the control group (Figure 2). However, the heterogeneity observed was substantial (I2=89), indicating that variability in effect size exists among these three studies (Figure 2). Each study employed different surgeons, sample sizes, and data collection techniques; therefore, the differences in these characteristics may potentially contribute to the variability [13,16,17]. Specifically, Simcox et al. indicated that a single orthopedic surgeon performed all the operations indicated within the study [17]. Furthermore, the limited number of studies utilized for this meta-analysis is a separate variable that may be influencing the heterogeneity.

Various sub-analyses, only analyzing two of the three studies, were conducted to observe differences in heterogeneity. The funnel plot, Figure 5, indicates that potential bias may exist within the Simcox et al. study, as the position of the point within the plot indicates an extremely positive effect size [17]. When comparing only Herzog et al. and Murakami et al., the p-value for the reduction in TAD for ADAPT versus the control group was no longer significant (Figure 3) [13,16]. However, the heterogeneity between these two studies was observed to be 47% (Figure 3) [13,16]. These findings suggest that Simcox et al. presented significant variability in the overall study [17]. The bias in Simcox et al. may emanate from performance bias, as a single fellowship-trained orthopedic trauma surgeon performed the procedures utilized within the study [17]. This limits randomization in individualized techniques among surgeons and influences the choice of treatment for individual patients. The latter may indicate a form of selection bias that may have occurred. However, the authors of this study attempted to mitigate this by controlling for fracture type and implant length within the analysis [17]. While present in all three studies, detection bias may have influenced the results in the Simcox et al. study, in comparison to the other two studies utilized [13,16,17]. Simcox et al., Murakami et al., and Herzog et al., all attempted to utilize a standardized method to analyze the radiographs; however, variation in positioning and interpretation of images raises some concerns [13,16,17].

In addition to analyzing the TAD between the ADAPT and control groups, Simcox et al., Murakami et al., and Herzog et al. assessed whether differences in operative time existed between these two cohorts [13,16,17]. Herzog et al. and Simcox et al. indicated that operative time was reduced in the procedures utilizing ADAPT; however, this difference was not significant, and the p-values in each study exceeded 0.01 [16,17]. On the other hand, Murakami et al. stipulated a significant decrease in operative time (p<0.05) in the ADAPT group versus the control group [13].

While more research must be performed to understand the true impact of computer-assisted navigation on operative time, these findings suggest the potential positive impact of ADAPT regarding this variable. Decreased operative times may reduce the surgical costs associated with prolonged anesthesia, risk of infection, and patient recovery time [23]. However, limitations to this finding do exist, as operative timing may possess a subjective component. Furthermore, screw position was not a variable analyzed within this meta-analysis. Screw position is a factor that can influence the success associated with osteosynthesis [24]. Ideal lag screw position, as stated by the articles, is characterized as center-center in both the anteroposterior and lateral radiographs [13,16,17].

The potential biases indicate the need for further research in assessing the overall impact of computer-assisted navigation in minimizing TAD. As computer-assisted technology is a fairly new development within the field of orthopedic surgery, limitations currently exist in analyzing its impact [10]. This is evident by the use of retrospective study designs in Herzog et al. and Simcox et al., with the former indicating the challenges associated with incorporating new technology into a surgical field [16,17]. However, the use of technological advancements is only expanding, as the application of computer-assisted surgery is immense; consequently, many more opportunities to assess the efficacy will be available [10]. 

Limitations associated with this meta-analysis include a low number of trials, varying heterogeneity, unstandardized data collection, and differing surgical techniques. The small sample size, bias, and varying population of patients may have contributed to the high heterogeneity in the study. Furthermore, as the ADAPT system is specific to the medical technology company, Stryker, potential bias may have existed in the formation of these articles. Nonetheless, numerous attempts at minimizing bias were considered to devise this article properly. While generalization may be subdued, the analysis results indicate ADAPT significantly decreased TAD compared to the control group. Future research, including a prospective study design with standardized data collection, a large sample size, multiple surgeons, and consistent surgical techniques across surgeons, may provide insight into this discussion.

## Conclusions

This meta-analysis investigated the impact of the computer-assisted navigation system, ADAPT, on the TAD distance of intramedullary nail fixation during internal fixation of an intertrochanteric fracture. All three of the studies displayed a statistically significant reduction in TAD for intramedullary nail fixation. The meta-analysis also examined the effect of the ADAPT system on operative time, in which two studies displayed a non-significant reduction and one study exhibited a significant decrease in operative time.

Our meta-analysis of three studies, including 193 subjects, demonstrated a reduction in TAD for intramedullary nail fixation using ADAPT computer-assisted navigation, with an average TAD distance 5.06 mm lower than the control group, which was statistically significant (p 0.01). The substantial heterogeneity observed (I2 =89%) signals variation in patient populations, surgeons, and methodology among the three studies. The results from this meta-analysis display the importance of utilizing computer-assisted navigation systems and the benefits that are brought to patient outcomes, including avoiding lag screw cut-outs. Overall, the authors of this meta-analysis suggest that computer-assisted navigation systems are beneficial in obtaining a reduction in TAD and believe further research is necessary to understand the implications, including operative time and possible improvements in screw position.
